# Supplementary material for: Complexome profiling of the Chlamydomonas psb28 mutant reveals TEF5 as an early PSII assembly factor
Source: Plant Cell. 2025 Mar 17;37(6):koaf055. doi: 10.1093/plcell/koaf055 (PMC12164590; doi:10.1093/plcell/koaf055)
Supplement: koaf055_Supplementary_Data [file koaf055_supplementary_data.zip › psb28_interactive.zip!/plots/intensity/qualityPlots/peptides/ELI2_Cre09.g393173.t1.1.html]

### Cre09.g393173.t1.1

Trivial name: ELI2  
SeqCoverage [median]: 33.70  
Mass: 18.75 kDa  
  
 

| Sequence | MissedCleavage | Mass [Da] | wtIntensity | muIntensity | Score |
| --- | --- | --- | --- | --- | --- |
| DTESARDVFAFGGSAPER | 1 | 1910.88 | 2563.05 | 6743.56 | 62.20 |
| DVFAFGGSAPER | 0 | 1251.59 | 26848.39 | 100986.74 | 165.18 |
| DVFAFGGSAPERVNGR | 1 | 1677.82 | 1198.22 | 1952.69 | 47.53 |
| ELHSVATSENLK | 0 | 1326.68 | 6585.62 | 21095.32 | 136.94 |
| LVSGVSLK | 0 | 801.50 | 8868.71 | 30598.19 | 144.38 |
| VAMMGFVSILGPELSK | 0 | 1677.88 | 5004.54 | 0.00 | 174.84 |
| VAMMGFVSILGPELSKK | 1 | 1805.98 | 0.00 | 3345.60 | 103.60 |
|  |  |  | 51068.5 | 164722.1 |  |
